# High-Resolution Chromosome Ideogram Representation of Currently Recognized Genes for Autism Spectrum Disorders

**DOI:** 10.3390/ijms16036464

**Published:** 2015-03-20

**Authors:** Merlin G. Butler, Syed K. Rafi, Ann M. Manzardo

**Affiliations:** Departments of Psychiatry & Behavioral Sciences and Pediatrics, University of Kansas Medical Center, Kansas City, KS 66160, USA; E-Mails: rafigene@yahoo.com (S.K.R.); amanzardo@kumc.edu (A.M.M.)

**Keywords:** high-resolution chromosome ideograms, autism, genetic evidence, autism spectrum disorders (ASD), ASD genes

## Abstract

Recently, autism-related research has focused on the identification of various genes and disturbed pathways causing the genetically heterogeneous group of autism spectrum disorders (ASD). The list of autism-related genes has significantly increased due to better awareness with advances in genetic technology and expanding searchable genomic databases. We compiled a master list of known and clinically relevant autism spectrum disorder genes identified with supporting evidence from peer-reviewed medical literature sources by searching key words related to autism and genetics and from authoritative autism-related public access websites, such as the Simons Foundation Autism Research Institute autism genomic database dedicated to gene discovery and characterization. Our list consists of 792 genes arranged in alphabetical order in tabular form with gene symbols placed on high-resolution human chromosome ideograms, thereby enabling clinical and laboratory geneticists and genetic counsellors to access convenient visual images of the location and distribution of ASD genes. Meaningful correlations of the observed phenotype in patients with suspected/confirmed ASD gene(s) at the chromosome region or breakpoint band site can be made to inform diagnosis and gene-based personalized care and provide genetic counselling for families.

## 1. Introduction

Classical autism or autistic disorder is common, with developmental difficulties noted by three years of age. It belongs to a group of heterogeneous conditions known as autism spectrum disorders (ASDs) with significant impairments in verbal and non-verbal communication and social interactions with restricted repetitive behaviors, specifically in movements and interests [1,2,3]. Other symptoms include lack of eye contact or focus, sleep disturbances and tactile defensiveness beginning at an early age. Several validated rating scales are used at a young age to help establish the diagnosis, including the autism diagnostic observation schedule (ADOS) and the autism diagnostic interview-revised (ADI-R) supported by pertinent medical history and clinical findings [4,5,6]. ASD affects about 1% of children in the general U.S. population with a 4:1 male to female ratio, usually without congenital anomalies or growth retardation [7,8].

Autism was first used as a term by Kanner in 1943 when describing a group of children lacking the ability to establish interpersonal contact and communication [9]. About one-fourth of children with autism are diagnosed by 2–3 years of age and show regression of skills in about 30% of cases. About 60% of ASD subjects show intellectual disabilities at a young age [10,11]. When comparing the prevalence of health disorders involving the central nervous system, autism ranks higher than epilepsy (6.5 cases per 1000), brain paralysis or dementia (2.5 cases/1000 for each) and Parkinson disease (two cases per 1000); genetic factors are related to many of these disorders [12,13]. Autism also occurs more commonly than congenital malformations in the general population, but dysmorphic findings are present in about 25% of children with autism. Microcephaly is seen in about 10% of cases, but macrocephaly is documented with larger frontal and smaller occipital lobes in about 20% of children with autism. Those with autism and extreme macrocephaly are at a greater risk to have *PTEN* tumor suppressor gene mutations [14], while another autism-related gene (*CHD8*) can also lead to macrocephaly and autism [15].

Autism is due to a wide range of genetic abnormalities, as well as non-genetic causes, including the environment, environmental and gene interaction (epigenetics) and metabolic disturbances (e.g., mitochondrial dysfunction), with the recurrence risk dependent on the family history and presence or absence of dysmorphic features. Candidate genes for ASD are identified by different means, including cytogenetic abnormalities (*i.e.*, translocations at chromosome breakpoints or deletions (e.g., the 22q11.2 deletion) indicating the location or loss of specific genes) in individuals with ASD along with overlapping linkage and functional data related to the clinical presentation, with certain chromosome regions identified by genetic linkage using DNA markers that co-inherit with the specific phenotype [16,17]. A representative example for such an occurrence is the proto-oncogene (MET) involved in pathways related to neuronal development [18] and found to be linked to the chromosome 7q31 band, where this gene is located. Decreased activity of the gene promoter was recognized when specific single nucleotide polymorphisms (SNPs) were present in this region by linkage studies. However, genetic linkage studies have received only limited success in the study of the genetics of autism. On the other hand, chromosomal microarray analysis using DNA probes disturbed across the genome can be used to detect chromosomal abnormalities at >100-times smaller than seen in high-resolution chromosome studies. Microarray studies have also become the first tier of genetic testing for this patient population and are recommended for all ASD patients [19]. Greater than 20% of studied patients with microarray analysis are found to have submicroscopic deletions or duplications in the genome containing genes that play a role in causing autism [20,21]. Identification of causative mutations is important to guide treatment selection and to manage medical co-morbidities, such as risks for seizures, developmental regression or for cancer (e.g., the *PTEN* gene).

Routine cytogenetic studies have shown abnormalities of chromosomes 2, 3, 4, 5, 7, 8, 11, 13, 15, 16, 17, 19, 22 and X, including deletions, duplications, translocations and inversions involving specific chromosome regions where known or candidate genes for ASD are located [22]. These studies further support the role of genetic factors in the causation of this common neurodevelopment disorder. Specifically, cytogenetic abnormalities involving the 15q11–q13 region are found in at least 1% of individuals with ASD and include *CYFIP1*, *GABRB3* and *UBE3A* genes in this chromosome region [23] and most recently the 15q11.2 BP1-BP2 microdeletion (Burnside-Butler) syndrome [24]. DNA copy number changes have also shown recurrent small deletions or duplications of the chromosome 16p11.2 band using microarray analysis [25,26] and the chromosome 15q13.2–q13.3 region [27], whereas copy number changes are noted throughout the genome in individuals with ASD, indicating the presence of multiple candidate genes on every human chromosome. These copy number changes are more often of the deletion type.

For idiopathic or non-syndromic autism, the empirical risk for siblings to be similarly affected is between 2% and 8% with an average of 4% [28]. In multiplex families having two or more affected children with autism, the recurrence risk may be as high as 25%, but generally ranges from 13% [29] to 19% [30] if due to single-gene disturbances as the cause, a major focus of this illustrative review. Advances in genetic technology beyond linkage or cytogenetic analysis of affected families with ASD or other complex disorders have led to genome-wide association studies (GWAS) involving hundreds of affected and control individuals by analyzing the distribution and clustering of hundreds and thousands of SNPs that have successfully been searched for candidate genes. The first GWAS for ASD was undertaken by Lauritsen *et al.* in 2006 [31] using 600 DNA markers in an isolated population of affected individuals from the Faroe Islands. They found an association of the chromosome 3p25.3 band, and later, other investigators studied more subjects with larger collections of genotyped markers and found several chromosome bands and regions ascertained when specific SNPs were over-represented in the ASD subjects, including 5p14.1, 5p15 and 16p13–p21 [32,33,34,35,36,37]. The studies implicated several gene families, including the cadherin family, encoding proteins for neuronal cell adhesion, while other genes (e.g., *SEMA5A*) were implicated in axonal guidance with lower gene expression levels in brain specimens from individuals with ASD [33], reviewed by Holt and Monaco [17]. Since that time, several additional studies searching for clinically relevant and known genes for ASD have identified a new collection of ASD genes [38,39,40,41,42,43,44,45,46,47,48,49,50,51,52,53].

The ability to identify an increased number of SNPs with advanced genetic platforms and extensive approaches using bioinformatics have led to improved access and a more thorough analysis. This has led to comparing genotyping data from GWAS and DNA copy number variants (CNVs) with the identification of structural genetic defects, such as submicroscopic deletions or duplications of the genome, which was not possible a few years ago. Separate studies using array comparative genomic hybridization or microarray analysis to investigate those individuals with ASD continue to yield useful data in identifying candidate genes for ASD in affected individuals [20,21,54]. The yield for microarray analysis is reported to be approximately 20% for identifying deletions or duplications at sites where known or candidate ASD genes are present. The use of more advanced technology, such as next-generation sequencing (whole genome or exome) will yield additional valuable information on the location and description of lesions of genes contributing to ASD with increasing evidence for specific and recurring mutations of single genes involved with neurodevelopment and function, leading to potential therapeutic discoveries and interventions.

Autism is frequent in single-gene conditions, such as fragile X syndrome, tuberous sclerosis, Rett syndrome or neurofibromatosis, but single-gene disorders as a whole account for less than 20% of all cases; therefore, most individuals with ASD are non-syndromic. The heritability of ASD, which takes into consideration the extent of genetic factors contributing to autism, is estimated to be as high as 90% [55]; hence the relevance and continued importance of investigating the role of genetics in the causation of ASD and expanded diagnostic testing to inform and guide treatment for individuals with identifiable genetic disturbances.

A current list of clinically relevant and known candidate genes for ASD is needed for diagnostic testing and genetic counselling purposes in the clinical setting. Historically, a previous list of known or candidate genes showing an association with ASD was reported in 2011 by Holt and Monaco [17] with the placement of 175 genes on chromosome ideograms. A much greater number of validated genes are now recognized as playing a pivotal role in ASD, warranting an updated, revised summary. We will utilize high-resolution chromosome ideograms (850 band level) to plot the location of genes now recognized by searching the literature and website information as playing a documented role in ASD. In tabular form, we will list the individual gene symbol, expanded name or description and chromosome location.

## 2. Results and Discussion

The diagnostic approach for an individual with ASD should include a clinical genetics evaluation with interviews of parents and health caregivers for the collection and overview of historical problems, a three-generation family pedigree, recording of developmental milestones and description of atypical behaviors along with medical and surgical procedures and a current list of medications and ongoing treatments. Laboratory tests should include lead, thyroid function, lactate and pyruvate levels in order to assess metabolic and mitochondrial functions that may be impacted by an underlying genetic disturbance along with cholesterol and urine collection for organic acid levels. Brain imaging and electroencephalogram patterns should be reviewed, if available. In addition, the ADI-R and ADOS instruments are used to test the diagnosis of ASD.

To further increase the diagnostic yield in individuals with ASD presenting for genetic service, Schaefer *et al.* [19] proposed and utilized a three-tier approach to include a genetic work-up by a clinical geneticist with expertise in dysmorphology to identify known syndromes with or without dysmorphic features (e.g., birth marks), growth anomalies (e.g., microcephaly, macrocephaly and short stature), viral titers (e.g., rubella) and metabolic screening (urine for organic acids and mucopolysaccharides, plasma lactate and amino acid levels). DNA testing for fragile X syndrome and Rett syndrome in females and males is also available, along with chromosomal and DNA microarrays to examine structural DNA lesions in those with a sporadic form of autism and the use of SNP arrays to examine for regions of homozygosity or uniparental disomy, whereby both members of a chromosome pair come from one parent [56]. Exome sequencing is now available particularly to those affected subjects with a positive family history of autism (multiplex families), if other diagnostic tests are uninformative. *PTEN* gene mutation screening would be indicated in those patients with extreme macrocephaly (head size > 2 SD) [14], if not previously done, and a review of brain MRI results. Serum and urine uric acid levels and assays for adenylate succinase deficiency should be done to include biochemical genetic studies and mitochondrial genome screening and function [57] if the above testing protocols are not diagnostic. Up to one in five children with ASD show findings of mitochondrial dysfunction [57], and a detailed genetic work-up will significantly increase the yield for the diagnosis of ASD, leading to a better understanding of causation, treatment and more accurate genetic counselling for those presenting for genetic services [20,21,54].

Advances made in genetic technology and bioinformatics have led to vastly improved genetic testing options for application in the clinical setting in patients presenting for genetic services [54]. Significant discoveries have been made with the recognition of genetic defects in the causation of ASD using microarray technology and, now, next generation sequencing. This technology has flourished with a combination of DNA probes used for both copy number variation and SNPs being required to identify segmental deletions and duplications in the genome and regions of homozygosity for the determination of identical by descent for the calculation of inbreeding coefficients or consanguinity status along with uniparental disomy of individual chromosomes [56].

Next generation exome DNA sequencing and RNA sequencing allows for discoveries of disease-causing genes and regulatory sequences required for normal function. Identifying and characterizing molecular signatures for novel or disturbed gene or exon expression and disease-specific profiles and patterns with expression heat maps have led to the recognition of interconnected disturbed gene pathways in many diseases, including a growing body of genetic evidence for autism and other psychiatric or aberrant behavioral disorders [54].

**Figure 1:**
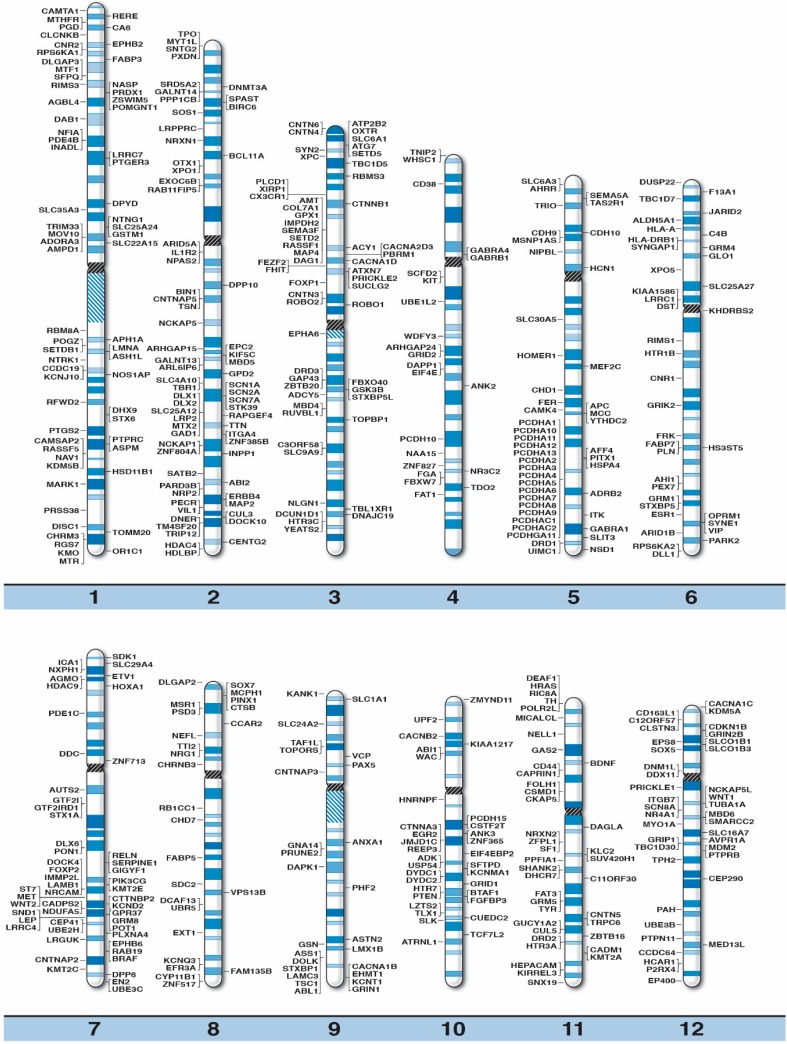

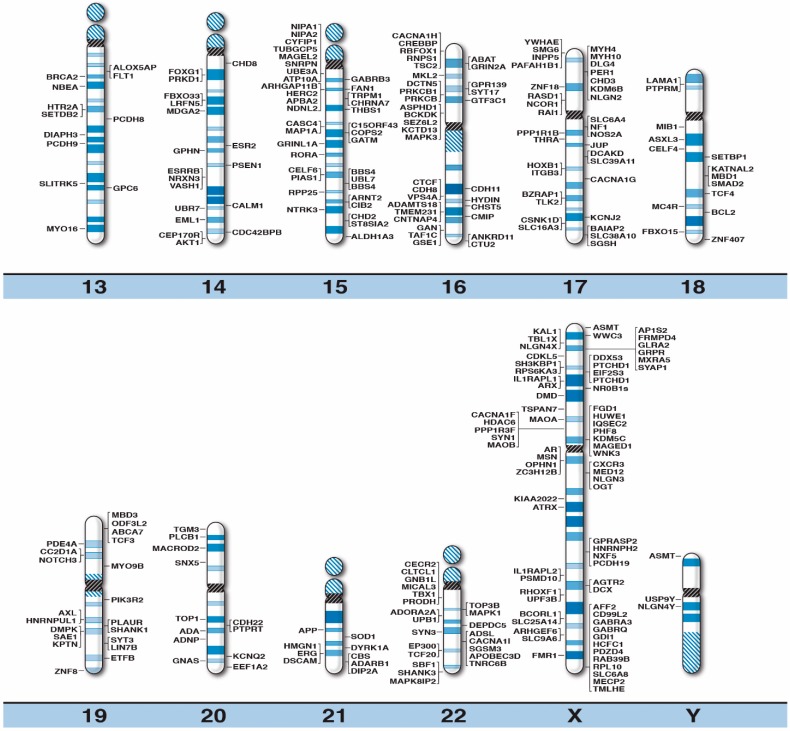
High-resolution human chromosome ideograms (850 band level) with the ASD gene symbol placed at the chromosomal band location. The centromere area, highlighted in black, separates the upper short “p” arm and lower long “q” arm for each chromosome. The gene symbols are arranged in alphabetical order with the expanded name and chromosome band position listed in Table 1.

The position for each known or candidate gene for ASD susceptibility is plotted on high-resolution chromosome ideograms (850 band level), as shown in Figure 1 below. We have included gene symbols and expanded names along with the chromosome band location in Table 1 for the 792 genes recognized as playing a role in ASD.

**Table 1:** Recognized genes for autism spectrum disorders (ASD) and their chromosome locations.

Gene Symbol	Gene Name	Location
*ABAT*	4-aminobutyrate aminotransferase	16p13.2
*ABCA7*	ATP-binding cassette, sub-family A (ABC1), member 7	19p13.3
*ABI1*	Abl-interactor 1	10p12.1
*ABI2*	Abl-interactor 2	2q33.2
*ABL1*	C-Abl oncogene 1, non-receptor tyrosine kinase	9q34.12
*ACY1*	Aminoacylase 1	3p21.2
*ADA*	Adenosine deaminase	20q13.12
*ADAMTS18*	A disintegrin-like and metalloproteinase with thrombospondin type 1 motif, 18	16q23.1
*ADARB1*	Adenosine deaminase, RNA-specific, B1	21q22.3
*ADCY5*	Adenylate cyclase 5	3q21.1
*ADK*	Adenosine kinase	10q22.2
*ADNP*	Activity-dependent neuroprotector homeobox	20q13.13
*ADORA2A*	Adenosine A2A receptor	22q11.23
*ADORA3*	Adenosine A3 receptor	1p13.2
*ADRB2*	Adrenergic, β 2 receptor	5q32
*ADSL*	Adenylosuccinate lyase	22q13.1
*AFF2*	AF4/fragile X mental retardation 2 (FMR2) family, member 2	Xq28
*AFF4*	AF4/fragile X mental retardation 2 (FMR2) family, member 4	5q31.1
*AGBL4*	ATP/GTP binding protein-like 4	1p33
*AGMO*	Alkylglycerol monooxygenase	7p21.1
*AGTR2*	Angiotensin II receptor, type 2	Xq23
*AHI1*	Abelson helper integration site 1	6q23.3
*AHRR*	Aryl hydrocarbon receptor repressor	5p15.33
*AKT1*	v-Akt murine thymoma viral oncogene homolog 1	14q32.33
*ALDH1A3*	Aldehyde dehydrogenase 1 family, member A3	15q26.3
*ALDH5A1*	Aldehyde dehydrogenase 5 family, member A1	6p22.3
*ALOX5AP*	Arachidonate 5-lipoxygenase-activating protein	13q12.3
*AMPD1*	Adenosine monophosphate deaminase 1	1p13.2
*AMT*	Aminomethyltransferase	3p21.31
*ANK2*	Ankyrin 2	4q25
*ANK3*	Ankyrin 3	10q21.2
*ANKRD11*	Ankyrin repeat domain 11	16q24.3
*ANXA1*	Annexin A1	9q21.13
*AP1S2*	Adaptor-related protein complex 1, sigma 2 subunit	Xp22.2
*APBA2*	Amyloid β precursor protein-binding, family A, member 2	15q13.1
*APC*	Adenomatosis polyposis coli	5q22.2
*APH1A*	APH1A γ secretase subunit	1q21.2
*APOBEC3D*	Apolipoprotein B mRNA editing enzyme, catalytic polypeptide-like 3D	22q13.1
*APP*	Amyloid β precursor protein	21q21.3
*AR*	Androgen receptor	Xq12
*ARHGAP11B*	Rho GTPase activating protein 11B	15q13.2
*ARHGAP15*	Rho GTPase activating protein 15	2q22.2
*ARHGAP24*	Rho GTPase activating protein 24	4q22.1
*ARHGEF6*	RAC/CDC42 guanine nucleotide exchange factor (GEF) 6	Xq26.3
*ARID1B*	AT rich interactive domain 1B (SWI1-like)	6q25.3
*ARID5A*	AT rich interactive domain 5A (MRF1-like)	2q11.2
*ARL6IP6*	ADP-ribosylation-like factor 6 interacting protein 6	2q23.3
*ARNT2*	Aryl-hydrocarbon receptor nuclear translocator 2	15q25.1
*ARX*	Aristaless related homeobox	Xp21.3
*ASH1L*	Ash1 (absent, small, or homeotic)-like (Drosophila)	1q22
*ASMT*	Acetylserotonin *O*-methyltransferase, X-chromosomal	Xp22.33
*ASMT*	Acetylserotonin *O*-methyltransferase, Y-chromosomal	Yp11.32
*ASPHD1*	Aspartate β-hydroxylase domain containing 1	16p11.2
*ASPM*	Asp (abnormal spindle) homolog, microcephaly associated	1q31.3
*ASS1*	Argininosuccinate synthetase	9q34.1
*ASTN2*	Astrotactin 2	9q33.1
*ASXL3*	Additional sex combs-like 3	18q12.1
*ATG7*	Autophagy related 7	3p25.3
*ATP10A*	ATPase, Class V, type 10A	15q11.2
*ATP2B2*	ATPase, Ca++ transporting, plasma membrane 2	3p25.3
*ATRNL1*	Attractin-like 1	10q25.3
*ATRX*	α thalassemia/mental retardation syndrome X-linked	Xq21.1
*ATXN7*	Ataxin 7	3p14.1
*AUTS2*	Autism susceptibility candidate 2	7q11.22
*AVPR1A*	Arginine vasopressin receptor 1A	12q14.2
*AXL*	AXL receptor tyrosine kinase	19q13.2
*BAIAP2*	BAI1-associated protein 2	17q25.3
*BBS4*	Bardet-Biedl syndrome 4	15q24.1
*BCKDK*	Branched chain ketoacid dehydrogenase kinase	16p11.2
*BCL11A*	B-Cell CLL/lymphoma 11A (zinc finger protein)	2p16.1
*BCL2*	B-cell CLL/lymphoma 2	18q21.33
*BCORL1*	Bc16 co-repressor-like 1	Xq26.1
*BDNF*	Brain-derived neurotrophic factor	11p14.1
*BIN1*	Bridging integrator 1	2q14.3
*BIRC6*	Baculoviral IAP repeat containing 6	2p22.3
*BRAF*	v-Raf murine sarcoma viral oncogene homolog B	7q34
*BRCA2*	Breast cancer 2, early onset	13q13.1
*BTAF1*	RNA polymerase II, B-TFIID transcription factor-associated, 170 kDa (Mot1 homolog, *S. cerevisiae*)	10q23.32
*BZRAP1*	Benzodiazepine receptor (peripheral) associated protein 1	17q23.2
*C11ORF30*	Chromosome 11 open reading frame 30	11q13.5
*C12ORF57*	Chromosome 12 open reading frame 57	12p13.31
*C15ORF43*	Chromosome 15 open reading frame 43	15q21.1
*C3ORF58*	Chromosome 3 open reading frame 58	3q24
*C4B*	Complement component 4B	6p21.33
*CA6*	Carbonic anhydrase VI	1p36.2
*CACNA1B*	Calcium channel, voltage-dependent, N type, α 1B subunit	9q34.3
*CACNA1C*	Calcium channel, voltage-dependent, L type, α 1C subunit	12p13.33
*CACNA1D*	Calcium channel, voltage-dependent, L type, α 1D subunit	3p14.3
*CACNA1F*	Calcium channel, voltage-dependent, α 1F subunit	Xp11.23
*CACNA1G*	Calcium channel, voltage-dependent, T type, α 1G subunit	17q21.33
*CACNA1H*	Calcium channel, voltage-dependent, α 1H subunit	16p13.3
*CACNA1I*	Calcium channel, voltage-dependent, T type, α 1I subunit	22q13.1
*CACNA2D3*	Calcium channel, voltage-dependent, α 2/δ subunit 3	3p21.1
*CACNB2*	Calcium channel, voltage-dependent, β 2 subunit	10p12.33
*CADM1*	Cell adhesion molecule 1	11q23.3
*CADPS2*	Ca++-dependent activator protein for secretion 2	7q31.32
*CALM1*	Calmodulin 1 (phosphorylase kinase, δ)	14q32.11
*CAMK4*	Calcium/calmodulin-dependent protein kinase	5q22.1
*CAMSAP2*	Calmodulin regulated spectrin-associated protein family, member 2	1q32.1
*CAMTA1*	Calmodulin binding transcription activator 1	1p36.31
*CAPRIN1*	Cell cycle associated protein 1	11p13
*CASC4*	Cancer susceptibility candidate 4	15q15.3
*CBS*	Cystathionine β-synthase	21q22.3
*CCAR2*	Cell cycle and apoptosis regulator 2	8p21.3
*CC2D1A*	Coiled-coil and C2 domain-containing 1A	19p13.12
*CCDC19*	Coiled-coil domain-containing protein 19	1q23.2
*CCDC64*	Coiled-coil domain-containing 64	12q24.23
*CD38*	CD38 molecule	4p15.32
*CD44*	CD44 molecule	11p13
*CD163L1*	CD163 molecule-like 1	12p13.31
*CD99L2*	CD99 molecule-like 2	Xq28
*CDC42BPB*	CDC42 binding protein kinase β (DMPK-like)	14q32.32
*CDH10*	Cadherin 10, type 2	5p14.2
*CDH22*	Cadherin-like 22	20q13.1
*CDH8*	Cadherin 8, type 2	16q22.1
*CDH9*	Cadherin 9, type 2	5p14.1
*CDH11*	Cadherin 11, type 2	16q21
*CDKL5*	Cyclin-dependent kinase-like 5	Xp22.13
*CDKN1B*	Cyclin-dependent kinase inhibitor 1B	12p13.1
*CECR2*	Cat eye syndrome chromosome region, candidate 2	22q11.21
*CELF4*	CUGBP, Elav-like family, member 4	18q12.2
*CELF6*	CUGBP, Elav-like family, member 6	15q23
*CENTG2*	Centaurin γ-2	2q37.2
*CEP170R*	Centrosomal protein 170B	14q32.33
*CEP290*	Centrosomal protein 290 kDa	12q21.32
*CEP41*	Centrosomal protein 41 kDa	7q32.2
*CHD1*	Chromodomain helicase DNA binding protein 1	5q21.1
*CHD2*	Chromodomain helicase DNA binding protein 2	15q26.1
*CHD3*	Chromodomain helicase DNA binding protein 3	17p13.1
*CHD7*	Chromodomain helicase DNA binding protein 7	8q12.2
*CHD8*	Chromodomain helicase DNA binding protein 8	14q11.2
*CHRM3*	Cholinergic receptor, muscarinic 3	1q43
*CHRNA7*	Cholinergic receptor, neuronal nicotinic, α 7	15q13.3
*CHRNB3*	Cholinergic receptor, neuronal nicotinic, β 3	8p11.21
*CHST5*	Carbohydrate sulfotransferase 5	16q22.3
*CIB2*	Calcium and integrin binding family member 2	15q25.1
*CKAP5*	Cytoskeleton associated protein 5	11p11.2
*CLCNKB*	Chloride channel voltage-sensitive kidney, B	1p36.13
*CLSTN3*	Calsyntenin 3	12p13.31
*CLTCL1*	Clathrin, heavy chain-like 1	22q11.21
*CMIP*	c-MAF inducing protein	16q23.2
*CNR1*	Cannabinoid receptor 1	6q15
*CNR2*	Cannabinoid receptor 2	1p36.11
*CNTN3*	Contactin 3	3p12.3
*CNTN4*	Contactin 4	3p26.3
*CNTN5*	Contactin 5	11q22.1
*CNTN6*	Contactin 6	3p26.3
*CNTNAP2*	Contactin associated protein-like 2	7q35
*CNTNAP3*	Contactin associated protein-like 3	9p13.1
*CNTNAP4*	Contactin associated protein-like 4	16q23.1
*CNTNAP5*	Contactin associated protein-like 5	2q14.3
*COL7A1*	Collagen, type VII, α 1	3p21.31
*COPS2*	Thyroid hormone receptor interactor 15	15q21.1
*CREBBP*	CREB binding protein	16p13.3
*CSMD1*	Cytoskeleton associated protein 5	11p11.2
*CSNK1D*	Casein kinase 1, δ	17q25
*CSTF2T*	Cleavage stimulation factor, 3' pre-RNA, subunit 2, 64 kDa, tau	10q21.1
*CTCF*	CCCTC-binding factor	16q22.1
*CTNNA3*	Catenin (cadherin-associated protein), α 3	10q21.3
*CTNNB1*	Catenin (cadherin-associated protein), β 1, 88 kDa	3p22.1
*CTSB*	Cathepsin B	8p23.1
*CTTNBP2*	Cortactin binding protein 2	7q31.31
*CTU2*	Cytosolic thiouridylase subunit 2 homolog (S. pombe)	16q24.3
*CUEDC2*	CUE domain containing 2	10q24.32
*CUL5*	Cullin 5	11q22.3
*CUL3*	Cullin 3	2q36.2
*CX3CR1*	Chemokine (C-X3-C motif) receptor 1	3p22.2
*CXCR3*	Chemokine, CXC motif, receptor 3	Xq13.1
*CYFIP1*	Cytoplasmic FMRP interacting protein 1	15q11.2
*CYP11B1*	Cytochrome P450, subfamily XIB, polypeptide 1	8q24.3
*DAB1*	Disabled homolog 1	1p32.2
*DAG1*	Dystroglycan 1 (dystrophin-associated glycoprotein 1)	3p21.31
*DAGLA*	Diacylglycerol lipase, α	11q12.2
*DAPK1*	Death-associated protein kinase 1	9q21.33
*DAPP1*	Dual adaptor of phosphotyrosine and 3-phosphoinositides 1	4q23
*DCAF13*	DDB1 and CUL4 associated factor 13	8q22.3
*DCAKD*	Dephospho-CoA kinase domain-containing protein	17q21.31
*DCTN5*	Dynactin 5	16p12.2
*DCUN1D1*	DCN1, domain containing protein 1	3q27.1
*DCX*	Doublecortin	Xq23
*DDC*	DOPA decarboxylase	7p12.1
*DDX11*	DEAD (Asp-Glu-Ala-Asp)/H box 11	12p11.21
*DDX53*	DEAD (Asp-Glu-Ala-Asp) box polypeptide 53	Xp22.11
*DEAF1*	DEAF1 transcription factor	11p15.5
*DEPDC5*	DEP domain containing 3 protein 5	22q12.2
*DHCR7*	7-dehydrocholesterol reductase	11q13.4
*DHX9*	DEAH (Asp-Glu-Ala-His) box helicase 9	1q25.3
*DIAPH3*	Diaphanous, Drosophila, homolog 3	13q21.2
*DIP2A*	DIP2 disco-interacting protein 2 homolog A (Drosophila)	21q22.3
*DISC1*	Disrupted in schizophrenia 1	1q42.2
*DLG4*	Discs, large, Drosophila, homolog 4	17p13.1
*DLGAP2*	Discs, large- associated protein 2	8p23.3
*DLGAP3*	Discs, large- associated protein 3	1p34.3
*DLL1*	δ-like 1 (Drosophila)	6q27
*DLX1*	Distal-less homeobox 1	2q31.1
*DLX2*	Distal-less homeobox 2	2q31.1
*DLX6*	Distal-less homeobox 6	7q21.3
*DMD*	Dystrophin	Xp21.1
*DMPK*	Dystrophia myotonica-protein kinase	19q13.32
*DNAJC19*	DNAJ Hsp40 homolog, subfamily C, member 19	3q26.33
*DNER*	δ- and notch-like epidermal growth factor-related receptor	2q36.3
*DNM1L*	Dynamin 1-like	12p11.21
*DNMT3A*	DNA (cytosine-5)-methyltransferase 3 α	2p23.3
*DOCK4*	Dedicator of cytokinesis 4	7q31.1
*DOCK10*	Dedicator of cytokinesis 10	2q36.2
*DOLK*	Dolichol kinase	9q34.1
*DPP10*	Dipeptidyl peptidase 10	2q14.1
*DPP6*	Dipeptidyl peptidase 6	7q36.2
*DPYD*	Dihydropyrimidine dehydrogenase	1p21.3
*DRD1*	Dopamine receptor D1	5q35.2
*DRD2*	Dopamine receptor D2	11q23.2
*DRD3*	Dopamine receptor D3	3q13.31
*DSCAM*	Down syndrome cell adhesion molecule	21q22.2
*DST*	Dystonin	6p12.1
*DUSP22*	Dual specificity phosphatase 22	6p25.3
*DYDC1*	DPY30 domain containing 1	10q23.1
*DYDC2*	DPY30 domain containing 2	10q23.1
*DYRK1A*	Dual-specificity tyrosine-phosphorylation-regulated kinase 1A	21q22.13
*EEF1A2*	Eukaryotic translation elongation factor 1 α 2	20q13.33
*EFR3A*	EFR3 homolog A (*S. cerevisiae*)	8q24.22
*EGR2*	Early growth response 2	10q21.3
*EHMT1*	Euchromatic histone methyltransferase 1	9q34.3
*EIF2S3*	Eukaryotic translation initiation factor 2, subunit 3 γ	Xp22.11
*EIF4E*	Eukaryotic translation initiation factor 4E	4q23
*EIF4EBP2*	Eukaryotic translation initiation factor 4E binding protein 2	10q22.1
*EML1*	Echinoderm microtubule associated protein like 1	14q32.2
*EN2*	Engrailed 2	7q36.3
*EP300*	E1A binding protein p300	22q13.2
*EP400*	E1A binding protein p400	12q24.33
*EPC2*	Enhancer of polycomb, Drosophila homolog of 2	2q23.1
*EPHA6*	Ephrin receptor A6	3q11.2
*EPHB2*	Ephrin receptor B2	1p36.12
*EPHB6*	Ephrin receptor B6	7q34
*EPS8*	Epidermal growth factor receptor pathway substrate 8	12p12.3
*ERBB4*	v-ERB-A avian erythroblastic leukemia viral oncogene homolog 4	2q34
*ERG*	v-ETS avian erythroblastosis virus E26 oncogene homolog	21q22.2
*ESR1*	Estrogen receptor 1	6q25.1
*ESR2*	Estrogen receptor 2	14q23.2
*ESRRB*	Estrogen-related receptor β	14q24.3
*ETFB*	Electron-transfer-flavoprotein, β polypeptide	19q13.41
*ETV1*	Ets variant 1	7p21.2
*EXOC6B*	Exocyst complex component 6B	2p13.2
*EXT1*	Exostosin 1	8q24.11
*F13A1*	Factor XIII, A1 subunit	6p25.1
*FABP3*	Fatty acid binding protein 3, muscle and heart (mammary-derived growth inhibitor)	1p35.2
*FABP5*	Fatty acid binding protein 5	8q21.13
*FABP7*	Fatty acid binding protein 7	6q22.31
*FAM135B*	Family with sequence similarity 135, member B	8q24.23
*FAN1*	FANCD2/FANCI-associated nuclease 1	15q13.2
*FAT1*	FAT tumor suppressor, Drosophila homolog of, 1	4q35.2
*FAT3*	FAT tumor suppressor, Drosophila homolog of , 3	11q14.3
*FBXO15*	F-box protein 15	18q22.3
*FBXO33*	F-box protein 33	14q21.1
*FBXO40*	F-box protein 40	3q13.33
*FBXW7*	F-box and WD repeat domain containing 7, E3 ubiquitin protein	4q31.3
*FER*	FPS/FES related tyrosine kinase	5q21.3
*FEZF2*	FEZ family zinc finger 2	3p14.2
*FGA*	Fibrinogen, A α polypeptide	4q31.3
*FGD1*	FYVE, Rho GEF and PH domain containing 1	Xp11.22
*FGFBP3*	Fibroblast growth factor binding protein 3	10q23.32
*FHIT*	Fragile histidine triad	3p14.2
*FLT1*	c-FMS-related tyrosine kinase 1	13q12.3
*FMR1*	Fragile X mental retardation 1 (FMR1)	Xq27.3
*FOLH1*	Folate hydrolase 1	11p11.2
*FOXG1*	Forkhead box G1	14q12
*FOXP1*	Forkhead box P1	3p13
*FOXP2*	Forkhead box P2	7q31.1
*FRK*	FYN-related kinase	6q22.1
*FRMPD4*	FERM and PDZ domain containing protein 4	Xp22.2
*GABRA1*	γ-aminobutyric acid A receptor, α 1	5q34
*GABRA3*	γ-aminobutyric acid receptor, α 3	Xq28
*GABRA4*	γ-aminobutyric acid receptor, α 4	4p12
*GABRB1*	γ-aminobutyric acid receptor, β 1	4p12
*GABRB3*	γ-aminobutyric acid receptor, β 3	15q12
*GABRQ*	γ-aminobutyric acid receptor, θ	Xq28
*GAD1*	Glutamate decarboxylase 1 (brain, 67 kDa)	2q31.1
*GALNT13*	UDP-*N*-acetyl-α-d-galactosamine:polypeptide *N*-acetylgalactosaminyl-transferase 13	2q23.3
*GALNT14*	UDP-*N*-acetyl-α-d-galactosamine:polypeptide *N*-acetylgalactosaminyl-transferase 14	2p23.1
*GAN*	Gigaxonin	16q24.1
*GAP43*	Growth associated protein 43	3q13.31
*GAS2*	Growth arrest-specific 2	11p14.3
*GATM*	Glycine amidinotransferase (l-arginine:glycine amidinotransferase)	15q21.1
*GDI1*	GDP dissociation inhibitor 1	Xq28
*GIGYF1*	GRB10 interacting GYF protein 1	7q22.1
*GLO1*	Glyoxalase I	6p21.2
*GLRA2*	Glycine receptor, α 2 subunit	Xp22.2
*GNA14*	Guanine nucleotide-binding protein, α 14	9q21.2
*GNAS*	Guanine nucleotide-binding protein, α-stimulating activity polypeptide I complex locus	20q13.32
*GNB1L*	Guanine nucleotide-binding protein, β 1-like	22q11.21
*GPC6*	Glypican 6	13q31.3
*GPD2*	Glycerol-3-phosphate dehydrogenase 2	2q24.1
*GPHN*	Gephyrin	14q23.3
*GPR139*	G protein-coupled receptor 139	16p12.3
*GPR37*	G protein-coupled receptor 37	7q31.33
*GPRASP2*	G protein-coupled receptor associated sorting protein 2	Xq22.1
*GPX1*	Glutathione peroxidase 1	3p21.31
*GRID1*	Glutamate receptor, ionotropic, δ 1	10q23.2
*GRID2*	Glutamate receptor, ionotropic, δ 2	4q22.1
*GRIK2*	Glutamate receptor, ionotropic, kainate 2	6q16.3
*GRIN1*	Glutamate receptor, ionotropic, *N*-methyl d-aspartate 1	9q34.3
*GRIN2A*	Glutamate receptor, ionotropic, *N*-methyl d-aspartate 2A	16p13.2
*GRIN2B*	Glutamate receptor, ionotropic, *N*-methyl d-aspartate 2B	12p13.1
*GRINL1A*	GRINL1A complex locus 1	15q21.3
*GRIP1*	Glutamate receptor interacting protein 1	12q14.3
*GRM1*	Glutamate receptor, metabotropic 1	6q24.3
*GRM4*	Glutamate receptor, metabotropic 4	6p21.31
*GRM5*	Glutamate receptor, metabotropic 5	11q14.3
*GRM8*	Glutamate receptor, metabotropic 8	7q31.33
*GRPR*	Gastrin-releasing peptide receptor	Xp22.2
*GSE1*	Gse1 coiled-coil protein	16q24.1
*GSK3B*	Glycogen synthase kinase 3 β	3q13.33
*GSN*	Gelsolin	9q33.2
*GSTM1*	Glutathione *S*-transferase M1	1p13.3
*GTF2I*	General transcription factor III	7q11.23
*GTF2IRD1*	GTF2I repeat domain containing 1	7q11.23
*GTF3C1*	General transcription factor IIIC, polypeptide 1, α	16p12.1
*GUCY1A2*	Guanylate cyclase 1, soluble, α 2	11q22.3
*HCAR1*	Hydroxycarboxylic acid receptor 1/G protein-coupled receptor 81	12q24.31
*HCFC1*	Host cell factor C1	Xq28
*HCN1*	Hyperpolarization activated cyclic nucleotide-gated potassium channel 1	5p12
*HDAC4*	Histone deacetylase 4	2q37.3
*HDAC6*	Histone deacetylase 6	Xp11.23
*HDAC9*	Histone deacetylase 9	7p21.1
*HDLBP*	High density lipoprotein binding protein	2q37.3
*HEPACAM*	Hepatic and glial cell adhesion molecule	11q24.2
*HERC2*	HECT domain and RCC1-like domain 2	15q13.1
*HLA-A*	Major histocompatibility complex, class I, A	6p22.1
*HLA-DRB1*	Major histocompatibility complex, class II, DR β 1	6p21.32
*HMGN1*	High mobility group nucleosome binding domain 1	21q22.2
*HNRNPF*	Heterogeneous nuclear ribonucleoprotein F	10q11.21
*HNRNPH2*	Heterogeneous nuclear ribonucleoprotein H2	Xq22.1
*HNRNPUL1*	Heterogeneous nuclear ribonucleoprotein U-like 1	19q13.2
*HOMER1*	Homer, Drosophila, homolog 1 of 1	5q14.1
*HOXA1*	Homeobox A1	7p15.3
*HOXB1*	Homeobox B1	17q21.32
*HRAS*	v-HA-RAS Harvey rat sarcoma viral oncogene homolog	11p15.5
*HS3ST5*	Heparan sulfate 3-*O-*sulfotransferase 5	6q22.31
*HSD11B1*	11-β-hydroxysteroid dehydrogenase type 1	1q32.2
*HSPA4*	Heat shock 70 kDa protein 4	5q31.1
*HTR1B*	5-hydroxytryptamine receptor 1B	6q14.1
*HTR2A*	5-hydroxytryptamine receptor 2A	13q14.2
*HTR3A*	5-hydroxytryptamine receptor 3A	11q23.2
*HTR3C*	5-hydroxytryptamine receptor 3, family member C	3q27.1
*HTR7*	5-hydroxytryptamine receptor 7	10q23.31
*HUWE1*	HECT, UBA and WWE domain containing 1, E3 ubiquitin protein ligase	Xp11.22
*HYDIN*	Hydrocephalus-inducing, mouse, homolog of	16q22.2
*ICA1*	Islet cell autoantigen 1	7p21.3
*IL1R2*	Interleukin 1 receptor, type II	2q11.2
*IL1RAPL1*	Interleukin 1 receptor accessory protein-like 1	Xp21.3
*IL1RAPL2*	Interleukin 1 receptor accessory protein-like 2	Xq22.3
*IMMP2L*	Inner mitochondrial membrane peptidase, subunit 2, *S. cerevisiae*, homolog of	7q31.1
*IMPDH2*	Inosine-5-prime monophosphate dehydrogenase 2	3p21.31
*INADL*	Inactivation no after-potential D-like	1p31.3
*INPP1*	Inositol polyphosphate-1-phosphatase	2q32.2
*INPP5*	Inositol polyphosphate-5-phosphatase	17p13.3
*IQSEC2*	IQ motif and Sec7 domain 2	Xp11.22
*ITGA4*	Integrin, α 4	2q31.3
*ITGB3*	Integrin, β 3	17q21.32
*ITGB7*	Integrin, β 7	12q13.13
*ITK*	IL20 inducible t-cell kinase	5q33.3
*JARID2*	Jumonji, AT rich interactive domain 2	6p22.3
*JMJD1C*	Jumonji domain containing 1C	10q21.3
*JUP*	Junction plakoglobin	17q21.2
*KAL1*	Kallmann syndrome interval 1	Xp22.31
*KANK1*	KN motif and ankyrin repeat domains 1	9p24.3
*KATNAL2*	Katanin p60 subunit A-like 2	18q21.1
*KCND2*	Potassium voltage-gated channel, Shal-related subfamily, member 2	7q31.31
*KCNJ2*	Potassium inwardly-rectifying channel, subfamily J, member 2	17q24.3
*KCNJ10*	Potassium inwardly-rectifying channel, subfamily J, member 10	1q23.2
*KCNMA1*	Potassium large conductance calcium-activated channel, subfamily M, α member 1	10q22.3
*KCNQ2*	Potassium voltage-gated channel, KQT-like subfamily, member 2	20q13.3
*KCNQ3*	Potassium voltage-gated channel, KQT-like subfamily, member 3	8q24.22
*KCNT1*	Potassium channel, subfamily T, member 1	9q34.3
*KCTD13*	Potassium channel tetramerization domain containing protein 13	16p11.2
*KDM5A*	Lysine (K)-specific demethylase 5A	12p13.33
*KDM5B*	Lysine (K)-specific demethylase 5B	1q32.1
*KDM5C*	Lysine (K)-specific demethylase 5C	Xp11.22
*KDM6B*	Lysine (K)-specific demethylase 6B	17p13.1
*KHDRBS2*	KH domain containing, RNA binding, signal transduction associated protein 2	6q11.1
*KIAA1217*	Sickle tail protein homolog	10p12.31
*KIAA1586*	KIAA1586	6p12.1
*KIAA2022*	KIAA2022	Xq13.3
*KIF5C*	Kinesin family member 5C	2q23.1
*KIRREL3*	Kin of IRRE like 3	11q24.2
*KIT*	v-KIT Hardy-Zuckerman 4 feline sarcoma viral oncogene homolog	4q12
*KLC2*	Kinesin light chain 2	11q13.2
*KMO*	Kynurenine 3-monooxygenase	1q43
*KMT2A*	Lysine (K)-specific methyltransferase 2A	11q23.3
*KMT2C*	Lysine (K)-specific methyltransferase 2C	7q36.1
*KMT2E*	Lysine (K)-specific methyltransferase 2E	7q22.3
*KPTN*	Kaptin (actin binding protein)	19q13.32
*LAMA1*	Laminin, α 1	18p11.23
*LAMB1*	Laminin, β 1	7q31.1
*LAMC3*	Laminin, γ 3	9q34.1
*LEP*	Leptin	7q32.1
*LIN7B*	Lin-7 homolog B (*C. elegans*)	19q13.33
*LMNA*	Lamin A/C	1q22
*LMX1B*	LIM homeobox transcription factor 1, β	9q33.3
*LRFN5*	Leucine-rich repeats and fibronectin type III domain containing 5	14q21.1
*LRGUK*	Leucine-rich repeats and guanylate kinase domain containing	7q33
*LRP2*	Low density lipoprotein receptor-related protein 2	2q31.1
*LRPPRC*	Leucine-rich PPR motif containing protein	2p21
*LRRC1*	Leucine-rich repeat-containing protein 1	6p12.1
*LRRC4*	Leucine-rich repeat-containing protein 4	7q32.1
*LRRC7*	Leucine-rich repeat-containing protein 7	1p31.1
*LZTS2*	Leucine zipper, putative tumor suppressor 2	10q24.31
*MACROD2*	Macro domain containing 2	20p12.1
*MAGED1*	Melanoma antigen family D, 1	Xp11.22
*MAGEL2*	MAGE-like 2	15q11.2
*MAOA*	Monoamine oxidase A	Xp11.3
*MAOB*	Monoamine oxidase B	Xp11.23
*MAP1A*	Microtubule-associated protein 1A	15q15.3
*MAP2*	Microtubule-associated protein (MAP) 2	2q34
*MAP4*	Microtubule-associated protein (MAP) 4	3p21.31
*MAPK1*	Mitogen-activated protein kinase 1	22q11.22
*MAPK3*	Mitogen-activated protein kinase 3	16p11.2
*MAPK8IP2*	Mitogen-activated protein kinase 8 interacting protein 2	22q13.33
*MARK1*	MAP/microtubule affinity-regulating kinase 1	1q41
*MBD1*	Methyl-CpG binding domain protein 1	18q21.1
*MBD3*	Methyl-CpG binding domain protein 3	19p13.3
*MBD4*	Methyl-CpG binding domain protein 4	3q21.3
*MBD5*	Methyl-CpG binding domain protein 5	2q23.1
*MBD6*	Methyl-CpG binding domain protein 6	12q13.2
*MC4R*	Melanocortin 4 receptor	18q21.32
*MCC*	Mutated in colorectal cancers	5q22.2
*MCPH1*	Microcephalin 1	8p23.1
*MDGA2*	Mephrin, A5 antigen, protein tyrosine phosphatase mu (MAM) domain containing glycosylphosphatidylinositol anchor 2	14q21.3
*MDM2*	MDM2 oncogene, E3 ubiquitin protein ligase	12q15
*MECP2*	Methyl CpG binding protein 2	Xq28
*MED12*	Mediator complex subunit 12	Xq13.1
*MED13L*	Mediator complex subunit 13-like	12q24.21
*MEF2C*	MADS box transcription myocyte enhancer factor 2, polypeptide C	5q14.3
*MET*	Met proto-oncogene	7q31.2
*MIB1*	Mind bomb E3 ubiquitin protein ligase 1	18q11.2
*MICAL3*	Microtubule-associated monooxygenase, calponin and lim domains-containing, 3	22q11.21
*MICALCL*	MICAL *C*-terminus-like protein	11p15.3
*MKL2*	Myocardin-like 2	16p13.12
*MOV10*	Moloney leukemia virus 10, mouse, homolog of	1p13.2
*MSN*	Moesin	Xq12
*MSNP1AS*	Moesin pseudogene 1 antisense	5p14.1
*MSR1*	Macrophage scavenger receptor	8p22
*MTF1*	Metal-regulatory transcription factor 1	1p34.3
*MTHFR*	5-10-methylene-tetrahydrofolate reductase	1p36.22
*MTR*	5-methyltetrahydrofolate-homocysteine *S*-methyltransferase	1q43
*MTX2*	Metaxin 2	2q31.1
*MXRA5*	Matrix-remodelling associated 5	Xp22.2
*MYH4*	Myosin, heavy chain 4, skeletal muscle	17p13.1
*MYH10*	Myosin, heavy chain 10, non-muscle	17p13.1
*MYO16*	Myosin XVI	13q33.3
*MYO1A*	Myosin IA	12q13.3
*MYO9B*	Myosin IXB	19p13.11
*MYT1L*	Myelin transcription factor 1-like	2p25.3
*NAA15*	*N*(α)-acetyltransferase 15, NatA auxiliary subunit	4q31.1
*NASP*	Nuclear autoantigenic sperm protein (histone-binding)	1p34.1
*NAV1*	Neuron navigator 1	1q32.1
*NBEA*	Neurobeachin	13q13.3
*NCKAP1*	NCK-associated protein 1	2q32.1
*NCKAP5*	NCK-associated protein 5	2q21.2
*NCKAP5L*	NCK-associated protein 5-like	12q13.12
*NCOR1*	Nuclear receptor corepressor 1	17p11.2
*NDNL2*	Necdin-like gene 2	15q13.1
*NDUFA5*	NADH-ubiquinone oxidoreductase 1 α subcomplex, 5	7q31.32
*NEFL*	Neurofilament protein, light polypeptide	8p21.2
*NELL1*	NEL-like 1	11p15.1
*NF1*	Neurofibromin 1	17q11.2
*NFIA*	Nuclear factor I/A	1p31.3
*NIPA1*	Non imprinted gene in Prader-Willi/Angelman syndrome chromosomal region 1	15q11.2
*NIPA2*	Non imprinted gene in Prader-Willi/Angelman syndrome chromosomal region 2	15q11.2
*NIPBL*	Nipped-B-like	5p13.2
*NLGN1*	Neuroligin 1	3q26.31
*NLGN2*	Neuroligin 2	17p13.1
*NLGN3*	Neuroligin 3	Xq13.1
*NLGN4X*	Neuroligin 4, X-linked	Xp22.31
*NLGN4Y*	Neuroligin 4, Y-linked	Yq11.221
*NOS1AP*	Nitric oxide synthase 1 (neuronal) adaptor protein	1q23.3
*NOS2A*	Nitric oxide synthase 2A	17q11.2
*NOTCH3*	Notch 3	19p13.12
*NPAS2*	Neuronal PAS domain protein 2	2q11.2
*NR0B1*	Nuclear receptor subfamily 0, group B, member 1	Xp21.2
*NR3C2*	Nuclear receptor subfamily 3, group C, member 2	4q31.23
*NR4A1*	Nuclear receptor subfamily 4, group A, member 1	12q13.13
*NRCAM*	Neuronal cell adhesion molecule	7q31.1
*NRG1*	Neuregulin 1	8p12
*NRP2*	Neuropilin 2	2q33.3
*NRXN1*	Neurexin I	2p16.3
*NRXN2*	Neurexin II	11q13.1
*NRXN3*	Neurexin III	14q24.3
*NSD1*	Nuclear receptor-binding Sa-var, enhancer of zeste, and trithorax domain protein 1	5q35.3
*NTNG1*	Netrin G1	1p13.3
*NTRK1*	Neurotrophic tyrosine kinase, receptor, type 1	1q23.1
*NTRK3*	Neurotrophic tyrosine kinase, receptor, type 3	15q25.3
*NXF5*	Nuclear RNA export factor 5	Xq22.1
*NXPH1*	Neurexophilin 1	7p21.3
*ODF3L2*	Outer dense fiber of sperm tails 3-like 2	19p13.3
*OGT*	O-linked *N*-acetylglucosamine transferase	Xq13.1
*OPHN1*	Oligophrenin 1	Xq12
*OPRM1*	Opioid receptor, mu 1	6q25.2
*OR1C1*	Olfactory receptor, family 1, subfamily C, member 1	1q44
*OTX1*	Orthodenticle Drosophila, homolog of	2p15
*OXTR*	Oxytocin receptor	3p25.3
*P2RX4*	Purinergic receptor P2X, ligand-gated ion channel, 4	12q24.31
*PAFAH1B1*	Platelet-activating factor acetylhydrolase 1B, regulatory subunit 1	17p13.3
*PAH*	Phenylalanine hydroxylase	12q23.2
*PARD3B*	PAR-3 family cell polarity regulator β	2q33.3
*PARK2*	Parkin	6q26
*PAX5*	Paired box 5	9p13.2
*PBRM1*	Polybromo 1	3p21.1
*PCDH10*	Protocadherin 10	4q28.3
*PCDH15*	Protocadherin 15	10q21.1
*PCDH19*	Protocadherin 19	Xq22.1
*PCDH8*	Protocadherin 8	13q14.3
*PCDH9*	Protocadherin 9	13q21.32
*PCDHA1*	Protocadherin α 1	5q31.3
*PCDHA10*	Protocadherin α 10	5q31.3
*PCDHA11*	Protocadherin α 11	5q31.3
*PCDHA12*	Protocadherin α 12	5q31.3
*PCDHA13*	Protocadherin α 13	5q31.3
*PCDHA2*	Protocadherin α 2	5q31.3
*PCDHA3*	Protocadherin α 3	5q31.3
*PCDHA4*	Protocadherin α 4	5q31.3
*PCDHA5*	Protocadherin α 5	5q31.3
*PCDHA6*	Protocadherin α 6	5q31.3
*PCDHA7*	Protocadherin α 7	5q31.3
*PCDHA8*	Protocadherin α 8	5q31.3
*PCDHA9*	Protocadherin α 9	5q31.3
*PCDHAC1*	Protocadherin α subfamily C, member 1	5q31.3
*PCDHAC2*	Protocadherin α subfamily C, member 2	5q31.3
*PCDHGA11*	Protocadherin γ subfamily A, member 11	5q31.3
*PDE1C*	Phosphodiesterase 1C	7p14.3
*PDE4A*	Phosphodiesterase 4A, cAMP-specific	19p13.2
*PDE4B*	Phosphodiesterase 4B, cAMP-specific	1p31.3
*PDZD4*	PDZ domain containing 4	Xq28
*PECR*	Peroxisomal trans-2-enoyl-CoA reductase	2q35
*PER1*	Period, Drosophila, homolog of	17p13.1
*PEX7*	Peroxisomal biogenesis factor 7	6q23.3
*PGD*	Phosphogluconate dehydrogenase	1p36.22
*PHF2*	PHD finger protein 2	9q22.31
*PHF8*	PHD finger protein 8	Xp11.22
*PIAS1*	Protein inhibitor of activated STAT, 1	15q23
*PIK3CG*	Phosphatidylinositol-3-kinase, catalytic, γ	7q22.3
*PIK3R2*	Phosphatidylinositol-3-kinase, regulatory subunit 2	19q13.11
*PINX1*	PIN2 interacting protein 1	8p23.1
*PITX1*	Paired-like homeodomain transcription factor 1	5q31.1
*PLAUR*	Plasminogen activator receptor, urokinase-type	19q13.31
*PLCB1*	Phospholipase C, β 1	20p12.3
*PLCD1*	Phospholipase C, δ 1	3p22.2
*PLN*	Phospholamban	6q22.31
*PLXNA4*	Plexin A4	7q32.3
*POGZ*	POGO transposable element with ZNF domain	1q21.3
*POLR2L*	Polymerase (RNA) II (DNA directed) polypeptide L, 7.6 kDa	11p15.5
*POMGNT1*	Protein *O*-mannose β-1, 2-*N*-acetylglucosaminyl-transferase	1p34.1
*PON1*	Paraoxonase 1	7q21.3
*POT1*	Protection of telomeres 1	7q31.33
*PPFIA1*	Protein tyrosine phosphatase, receptor type, F polypeptide, interacting protein, α 1	11q13.3
*PPP1CB*	Protein phosphatase 1, catalytic subunit, β isozyme	2p23.2
*PPP1R1B*	Protein phosphatase 1, regulatory (inhibitor) subunit 1B	17q12
*PPP1R3F*	Protein phosphatase 1, regulatory (inhibitor) subunit 3F	Xp11.23
*PRODH*	Proline dehydrogenase (oxidase) 1	22q11.21
*PRICKLE1*	Prickle, Drosophila, homolog of, 1	12q12
*PRICKLE2*	Prickle, Drosophila, homolog of, 2	3p14.1
*PRKCB*	Protein kinase C, β	16p12.2
*PRKCB1*	Protein kinase C, β-1	16p12.2
*PRKD1*	Protein kinase D1	14q12
*PRDX1*	Peroxiredoxin 1	1p34.1
*PRSS38*	Protease, serine, 38	1q42.13
*PRUNE2*	Prune, Drosophila, homolog of, 2	9q21.2
*PSD3*	Pleckstrin and Sec7 domains-containing protein 3	8p22
*PSEN1*	Presenilin 1	14q24.2
*PSMD10*	Proteasome 26S subunit, non-ATPase, 10	Xq22.3
*PTCHD1*	Patched domain containing protein 1	Xp22.11
*PTEN*	Phosphatase and tensin homolog	10q23.31
*PTGER3*	Prostaglandin E receptor 3, EP3 subtype	1p31.1
*PTGS2*	Prostaglandin-endoperoxide synthase 2	1q31.1
*PTPN11*	Protein tyrosine phosphatase, non-receptor type 11	12q24.13
*PTPRB*	Protein tyrosine phosphatase, receptor type, B	12q15
*PTPRC*	Protein tyrosine phosphatase, receptor type, C	1q31.3
*PTPRM*	Protein tyrosine phosphatase, receptor type, M	18p11.23
*PTPRT*	Protein tyrosine phosphatase, receptor type, T	20q13.11
*PXDN*	Peroxidasin, Drosophila homolog of	2p25.3
*RAB11FIP5*	RAB11 family-interacting protein 5	2p13.2
*RAB19*	RAB19, member RAS oncogene family	7q34
*RAB39B*	RAS-associated protein RAB39B	Xq28
*RAI1*	Retinoic acid induced gene 1	17p11.2
*RAPGEF4*	Rap guanine nucleotide exchange factor	2q31.1
*RASD1*	RAS protein, dexamethasone-induced, 1	17p11.2
*RASSF1*	RAS association (ralGDS/AF-6) domain family member 1	3p21.31
*RASSF5*	RAS association domain family protein 5	1q32.1
*RB1CC1*	RB1-inducible coiled-coil 1	8q11.23
*RBFOX1*	RNA binding protein FOX-1, *C. elegans*, homolog of, 1	16p13.3
*RBM8A*	RNA binding motif protein 8A	1q21.1
*RBMS3*	RNA binding motif protein, single stranded interacting, 3	3p24.1
*REEP3*	Receptor expression-enhancing protein 3	10q21.3
*RELN*	Reelin	7q22.1
*RERE*	RE-repeats encoding gene	1p36.23
*RFWD2*	Ring finger and WD repeat domains-containing protein 2	1q25.2
*RGS7*	Regulator of G protein signaling 7	1q43
*RHOXF1*	RHOX homeobox family, member 1	Xq24
*RIC8A*	RIC8 guanine nucleotide exchange factor A	11p15.5
*RIMS1*	Regulating synaptic membrane exocytosis 1	6q13
*RIMS3*	Protein regulating synaptic membrane exocytosis 3	1p34.2
*RNPS1*	RNA binding protein S1	16p13.3
*ROBO1*	Roundabout, Drosophila, homolog of, 1	3p12.2
*ROBO2*	Roundabout, Drosophila, homolog of, 2	3p12.3
*RORA*	RAR-related orphan receptor A	15q22.2
*RPL10*	Ribosomal protein L10	Xq28
*RPP25*	Ribonuclease P/MRP 25 kDa subunit	15q24.2
*RPS6KA1*	Ribosomal protein S6 kinase, 90 kDa, polypeptide 1	1p36.11
*RPS6KA2*	Ribosomal protein S6 kinase, 90 kDa, polypeptide 2	6q27
*RPS6KA3*	Ribosomal protein S6 kinase, 90 kDa, polypeptide 3	Xp22.12
*RUVBL1*	RuvB-E. coli, homolog-like 1	3q21.3
*SAE1*	SUMO1 activating enzyme, subunit 1	19q13.32
*SATB2*	Special AT-rich sequence-binding protein 2	2q33.1
*SBF1*	SET binding factor 1	22q13.33
*SCFD2*	Sec1 family domain containing 2	4q12
*SCN1A*	Sodium channel, neuronal, type I, α subunit	2q24.3
*SCN2A*	Sodium channel, voltage-gated, type II, α subunit	2q24.3
*SCN7A*	Sodium channel, voltage-gated, type VII, α subunit	2q24.3
*SCN8A*	Sodium channel, voltage-gated, type VIII, α subunit	12q13.13
*SDC2*	Syndecan 2	8q22.1
*SDK1*	Sidekick cell adhesion molecule 1	7p22.2
*SEMA3F*	Sema domain, immunoglobulin domain (Ig), short basic domain, secreted, (semaphorin) 3F	3p21.31
*SEMA5A*	Semaphorin 5A	5p15.31
*SERPINE1*	Serpin peptidase inhibitor, clade E (nexin, plasminogen activator inhibitor type 1), member 1	7q22.1
*SETBP1*	SET binding protein 1	18q12.3
*SETD2*	SET domain containing protein 2	3p21.31
*SETD5*	SET domain containing protein 5	3p25.3
*SETDB1*	SET domain, bifurcated, 1	1q21.3
*SETDB2*	SET domain, bifurcated, 2	13q14.2
*SEZ6L2*	Seizure related 6 homolog (mouse)-like 2	16p11.2
*SF1*	Splicing factor 1	11q13.1
*SFPQ*	Splicing factor proline/glutamine-rich	1p34.3
*SFTPD*	Surfactant, pulmonary-associated protein D	10q22.3
*SGSH*	*N*-sulfoglucosamine sulfohydrolase	17q25.3
*SGSM3*	Small G protein signaling modulator 3	22q13.1
*SH3KBP1*	SH3-domain kinase binding protein 1	Xp22.12
*SHANK1*	SH3 and multiple ankyrin repeat domains 1	19q13.3
*SHANK2*	SH3 and multiple ankyrin repeat domains 2	11q13.4
*SHANK3*	SH3 and multiple ankyrin repeat domains 3	22q13.33
*SLC16A3*	Solute carrier family 16 (monocarboxylic acid transporter), member 3	17q25
*SLC16A7*	Solute carrier family 16 (monocarboxylic acid transporter), member 7	12q14.1
*SLC1A1*	Solute carrier family 1 (neuronal/epithelial high affinity glutamate transporter), member 1	9p24.2
*SLC22A15*	Solute carrier family 22, (organic cation transporter), member 15	1p13.1
*SLC24A2*	Solute carrier family 24 (sodium/potassium/calcium exchanger), member 2	9p22.1
*SLC25A12*	Solute carrier family 25 (mitochondrial carrier, Aralar), member 12	2q31.1
*SLC25A14*	Solute carrier family 25 (mitochondrial carrier, brain), member 14	Xq26.1
*SLC25A24*	Solute carrier family 25 (mitochondrial carrier, phosphate carrier), member 24	1p13.3
*SLC25A27*	Solute carrier family 25, member 27	6p12.3
*SLC29A4*	Solute carrier family 29 (equilibrative nucleoside transporter), member 4	7p22.1
*SLC30A5*	Solute carrier family 30 (zinc transporter), member 5	5q13.1
*SLC35A3*	Solute carrier family 35 (UDP-*N*-acetylglucosamine transporter), member 3	1p21.2
*SLC38A10*	Solute carrier family 38, member 10	17q25.3
*SLC39A11*	Solute carrier family 39 (metal ion transporter), member 11	17q21.31
*SLC4A10*	Solute carrier family 4 (sodium bicarbonate transporter-like), member 10	2q24.2
*SLC6A1*	Solute carrier family 6 (neurotransmitter transporter), member 1	3p25.3
*SLC6A3*	Solute carrier family 6 (neurotransmitter transporter, dopamine), member 3	5p15.33
*SLC6A4*	Solute carrier family 6 (neurotransmitter transporter, serotonin), member 4	17q11.2
*SLC6A8*	Solute carrier family 6 (neurotransmitter transporter, creatine), member 8	Xq28
*SLC9A6*	Solute carrier family 9 (sodium/hydrogen exchanger), member 6	Xq26.3
*SLC9A9*	Solute carrier family 9 (sodium/hydrogen exchanger), member 9	3q24
*SLCO1B1*	Solute carrier organic anion transporter family, member 1B1	12p12.2
*SLCO1B3*	Solute carrier organic anion transporter family, member 1B3	12p12.2
*SLIT3*	Slit, Drosophila, homolog of, 3	5q35.1
*SLITRK5*	SLIT and NTRK-like family, member 5	13q31.2
*SLK*	STE20-like kinase	10q24.33
*SMAD2*	SMAD family member 2	18q21.1
*SMARCC2*	SWI/SNF related, matrix associated, actin dependent regulator of chromatin, subfamily C, member 2	12q13.2
*SMG6*	SMG 6, *C. elegans*, homolog of	17p13.3
*SND1*	EBNA2 coactivator p100	7q32.1
*SNRPN*	Small nuclear ribonucleoprotein polypeptide N	15q11.2
*SNTG2*	Syntrophin, γ 2	2p25.3
*SNX19*	Sorting nexin 19	11q25
*SNX5*	Sorting nexin 5	20p11.23
*SOD1*	Superoxide dismutase 1, soluble	21q22.11
*SOS1*	Son of sevenless (SOS), Drosophila, homolog 1	2p22.1
*SOX5*	SRY (sex determining region Y)-box 5	12p12.1
*SOX7*	SRY (sex determining region Y)-box 7	8p23.1
*SPAST*	Spastin	2p22.3
*SRD5A2*	Steroid-5-α-reductase, 2	2p23.1
*ST7*	Suppressor of tumorigenicity 7	7q31.2
*ST8SIA2*	ST8 α-*N*-acetyl-neuraminide α-2,8-sialyltransferase 2	15q26.1
*STK39*	Serine/threonine protein kinase 39	2q24.3
*STX6*	Syntaxin 6	1q25.3
*STX1A*	Syntaxin 1A	7q11.23
*STXBP1*	Syntaxin-binding protein 1	9q34.1
*STXBP5*	Syntaxin-binding protein 5	6q24.3
*STXBP5L*	Syntaxin-binding protein 5-like	3q13.33
*SUCLG2*	Succinate-CoA ligase, GDP-forming, β subunit	3p14.1
*SUV420H1*	Suppressor of variegation 4–20, Drosophila, homolog of, 1	11q13.2
*SYAP1*	Synapse associated protein 1	Xp22.2
*SYN1*	Synapsin 1	Xp11.23
*SYN2*	Synapsin II	3p25.2
*SYN3*	Synapsin III	22q12.3
*SYNE1*	Spectrin repeat containing nuclear envelope 1	6q25.2
*SYNGAP1*	Synaptic RAS-GTPase-activating protein 1	6p21.32
*SYT17*	Synaptotagmin XVII	16p12.3
*SYT3*	Synaptotagmin III	19q13.33
*TAF1C*	TATA box-binding protein-associated factor 1C	16q24.1
*TAF1L*	TATA box-binding protein-associated factor 1-like	9p21.1
*TAS2R1*	Taste receptor, type 2, member 1	5p15.31
*TBC1D30*	TBC1 domain family, member 30	12q14.3
*TBC1D5*	TBC1 domain family, member 5	3p24.3
*TBC1D7*	TBC1 domain family, member 7	6p24
*TBL1X*	Transducin-β-like 1, X-linked	Xp22.31
*TBL1XR1*	Transducin-β-like 1 receptor 1	3q26.32
*TBR1*	T-box, brain, 1	2q24.2
*TBX1*	T-box 1	22q11.21
*TCF3*	Transcription factor 3	19p13.3
*TCF4*	Transcription factor 4	18q21.2
*TCF20*	Transcription factor 20 (AR1)	22q13.2
*TCF7L2*	Transcription factor 7-like 2 (t-cell specific, HMG-box)	10q25.2
*TDO2*	Tryptophan 2,3-dioxygenase	4q32.1
*TGM3*	Transglutaminase 3	20p13
*TH*	Tyrosine hydroxylase	11p15.5
*THBS1*	Thrombospondin 1	15q14
*THRA*	Thyroid hormone receptor, α-1	17q21.1
*TLK2*	Tousled-like kinase 2	17q23.2
*TLX1*	T-cell leukemia homeobox 1	10q24.31
*TM4SF20*	Transmembrane 4 L6 family, member 20	2q36.3
*TMEM231*	Transmembrane protein 231	16q23.1
*TMLHE*	Epsilon-trimethyllysine hydroxylase	Xq28
*TNIP2*	TNFAIP3 interacting protein 2	4p16.3
*TNRC6B*	Trinucleotide repeat containing 6B	22q13.1
*TOMM20*	MAS20P, *S. cerevisiae*, homolog of	1q42.3
*TOP1*	Topoisomerase, DNA, I	20q12
*TOP3B*	Topoisomerase, DNA, III, β	22q11.22
*TOPBP1*	Topoisomerase (DNA) II-binding protein 1	3q22.1
*TOPORS*	Topoisomerase I-binding, arginine/serine-rich, E3 ubiquitin protein ligase	9p21.1
*TPH2*	Tryptophan hydroxylase 2	12q21.1
*TPO*	Thyroid peroxidase	2p25.3
*TRIM33*	Tripartite motif containing protein 33	1p13.2
*TRIO*	Trio Rho guanine nucleotide exchange factor	5p15.2
*TRIP12*	Thyroid hormone receptor interactor 12	2q36.3
*TRPC6*	Transient receptor potential cation channel, subfamily C, member 6	11q22.1
*TRPM1*	Transient receptor potential cation channel, subfamily M, member 1	15q13.3
*TSC1*	Tuberous sclerosis 1	9q34.1
*TSC2*	Tuberous sclerosis 2	16p13.3
*TSN*	Translin	2q14.3
*TSPAN7*	Tetraspanin 7	Xp11.4
*TTI2*	TELO2-interacting protein 2	8p12
*TTN*	Titin	2q31.2
*TUBA1A*	Tubulin, α-1A	12q13.12
*TUBGCP5*	Tubulin-γ complex-associated protein 5	15q11.2
*TYR*	Tyrosinase	11q14.3
*UBE1L2*	Ubiquitin-activating enzyme, E1-like 2	4q13.2
*UBE2H*	Ubiquitin-conjugating enzyme E2H	7q32.2
*UBE3A*	Ubiquitin protein ligase E3A	15q11.2
*UBE3B*	Ubiquitin protein ligase E3B	12q24.11
*UBE3C*	Ubiquitin protein ligase E3C	7q36.3
*UBL7*	Ubiquitin-like 7	15q24.1
*UBR5*	Ubiquitin protein ligase E3 component *N*-recognin 5	8q22.3
*UBR7*	Ubiquitin protein ligase E3 component *N*-recognin 7	14q32.12
*UIMC1*	Ubiquitin interaction motif containing 1	5q35.2
*UPB1*	Ureidopropionase, β 1	22q11.23
*UPF2*	UPF2, yeast, homolog of	10p14
*UPF3B*	UPF3, yeast, homolog of, B	Xq24
*USP54*	Ubiquitin specific peptidase 54	10q22.2
*USP9Y*	Ubiquitin specific protease 9, Y-chromosome	Yq11.21
*VASH1*	Vasohibin 1	14q24.3
*VCP*	Valosin containing protein	9p13.3
*VIL1*	Villin 1	2q35
*VIP*	Vasoactive intestinal peptide (VIP)	6q25.2
*VPS13B*	Vacuolar protein sorting 13, yeast, homolog of, B	8q22.2
*VPS4A*	Vacuolar protein sorting 4 homolog A (*S. cerevisiae*)	16q22.1
*WAC*	WW domain containing adaptor with coiled-coil	10p12.1
*WDFY3*	WD repeat and FYVE domain containing 3	4q21.23
*WHSC1*	Wolf-Hirschhorn syndrome candidate 1	4p16.3
*WNK3*	Protein kinase lysine deficient 3	Xp11.22
*WNT1*	Wingless-type MMTV integration site family, member 1	12q13.12
*WNT2*	Wingless-type MMTV integration site family, member 2	7q31.2
*WWC3*	WWC family member 3	Xp22.32
*XIRP1*	Cardiomyopathy-associated protein 1	3p22.2
*XPC*	Xeroderma pigmentosum complementation group C	3p25.1
*XPO1*	Exportin 1	2p15
*XPO5*	Exportin 5	6p21.1
*YEATS2*	YEATS domain containing 2	3q27.1
*YTHDC2*	YTH domain containing 2	5q22.2
*YWHAE*	Tyrosine 3-monooxygenase, tryptophan 5-monooxygenase activation protein, epsilon isoform	17p13.3
*ZBTB16*	Zinc finger- and BTB domain-containing protein 16	11q23.1
*ZBTB20*	Zinc finger- and BTB domain-containing protein 20	3q13.31
*ZC3H12B*	Zinc finger CCCH domain-containing protein 12B	Xq12
*ZFPL1*	Zinc finger protein-like 1	11q13.1
*ZMYND11*	Zinc finger, MYND-type containing 11	10p15.3
*ZNF18*	Zinc finger protein 18	17p12
*ZNF365*	Zinc finger protein 365	10q21.2
*ZNF385B*	Zinc finger protein 385B	2q31.3
*ZNF407*	Zinc finger protein 407	18q23
*ZNF517*	Zinc finger protein 517	8q24.3
*ZNF8*	Zinc finger protein 8	19q13.43
*ZNF713*	Zinc finger protein 713	7p11.2
*ZNF804A*	Zinc finger protein 804A	2q32.1
*ZNF827*	Zinc finger protein 827	4q31.22
*ZSWIM5*	Zinc finger, SWIM-type containing 5	1p34.1

## 3. Experimental Section

We used computer-based internet websites and PubMed (https://www.ncbi.nlm.nih.gov/pubmed) to search key words for genetics and autism. This included the integrated catalogue of human genetic studies related to autism found at the Simons Foundation Autism Research Initiative (SFARI) website (https://gene.sfari.org), which currently lists 667 genes reported as of 25 February 2015. This public access initiative is an ongoing curated collection of clinically proven ASD genes supported by clinical and autism experts, medical geneticists and laboratory specialists in the study of autism. This site includes gene description and evidence of support for causation with cited literature reports. We examined peer-reviewed articles found in the medical literature following our search for genetic evidence (*i.e.*, gene variants, mutations or disturbed gene function) and the involvement of genetics playing a role in autism. Sources included whole-genome sequencing of ASD families randomly selected with at least one unaffected sibling [40] or gene expression profiles in ASD [39] along with other informative websites (e.g., Online Mendelian Inheritance in Man, www.OMIM.org). We then compiled the list of genes from these major sources for a total of 792 genes, whereby at least one mechanism was involved for each gene that could lead to ASD, a heterogeneous condition involving many genes; as our report is focused on the compilation of ASD genes from peer-reviewed research articles and authoritative computer website genomic databases for autism and not necessarily related to causal relationships between the individual gene and ASD. Those genes recognized, to date, as playing a role in ASD susceptibility and causation generally appear to impact chromatin remodeling, metabolism, mRNA translation, cell adhesion and synaptic function [39].

SFARI is a publicly available manually curated web-based searchable site of human genes with links to ASD and includes genes in catalogue form based on five categories—genetic association, syndromic, rare single-gene variant and functional and multi-genetic copy number variation—supported by cited research publications for each. Additional literature sources in our study consisted of both primary research articles and reviews summarizing genetic evidence. Many of the listed genes were identified in multiple research studies and widely reported in literature reviews, data repositories and/or computer genomic-based websites for autism (e.g., SFARI). A large number of genes showed a varied relationship to autism and neurodevelopment, but the mass of the literature surveyed limits the reliability of our relative strength estimates for the ASD and gene associations. The gene would be included if cited and recognized in peer-reviewed publications (e.g., PubMed) with supportive genetic evidence (e.g., genetic linkage, GWAS, functional gene expression patterns, informative SNPs, CNVs or identified gene mutations). Other supporting genetic evidence can be found at Simons Foundation Autism Research Initiative (SFARI) at https://sfari.org/sfari-initiatives/simons-simplex-collection, the National Institutes of Health (NIH) at https://www.ncbi.nlm.nih.gov/gap, the Online Inheritance in Man (OMIM) at www.omim.org or Genecards at https://www.genecards.org.

## 4. Conclusions

Readily available tissue sources, such as peripheral blood, established lymphoblastoid cell lines and saliva, hold promise for more advances in ASD by enabling the identification of new genes and a better understanding of the causation and disease mechanisms to further stimulate research with the hope to discover new treatment modalities impacted by the recognition of known disease-causing or candidate genes for ASD. We illustrated the master list of clinically relevant and known ASD genes in our summary by plotting individual genes on high-resolution chromosome ideograms and generated a tabular form to increase the awareness required for genetic testing and counselling purposes for family members presenting for genetic services. Creating a master list of genes related to ASD is a complicated process; new genes are continually identified, but not all genes are equally important or certain to be causative. Additional research is needed to further investigate the causal relationships between the specific gene and ASD. The authors encourage the use of this collection of known and clinically relevant candidate genes for ASD in their evaluation of patients and families presenting for genetic testing options and for accurate genetic counselling.
